# Optical properties of hybrid T3Pyr/SiO_2_/3C-SiC nanowires

**DOI:** 10.1186/1556-276X-7-680

**Published:** 2012-12-17

**Authors:** Filippo Fabbri, Francesca Rossi, Manuela Melucci, Ilse Manet, Giovanni Attolini, Laura Favaretto, Massimo Zambianchi, Giancarlo Salviati

**Affiliations:** 1 IMEM-CNR Institute, Parco Area delle Scienze 37/A, Parma, 43124, Italy; 2 ISOF-CNR Institute, via P. Gobetti, 101, Bologna, 40129, Italy

**Keywords:** Nanowires, Functionalization, Silicon carbide, Oligothiophene, Fluorescence, 81.07.Pr78.67.Uh

## Abstract

A new class of nanostructured hybrid materials is developed by direct grafting of a model thiophene-based organic dye on the surface of 3C-SiC/SiO_2_ core/shell nanowires. TEM-EDX analysis reveals that the carbon distribution is more spread than it would be, considering only the SiC core size, suggesting a main contribution from C of the oligothiophene framework. Further, the sulfur signal found along the treated wires is not detected in the as-grown samples. In addition, the fluorescent spectra are similar for the functionalized nanostructures and T3Pyr in solution, confirming homogeneous molecule grafting on the nanowire surface. Chemical and luminescence characterizations confirm a homogeneous functionalization of the nanowires. In particular, the fluorophore retains its optical properties after functionalization.

## Background

Organic functionalization is emerging as an important area in the development of new semiconductor-based materials and devices. Direct immobilization of molecules or other types of selective receptors onto a semiconductor surface allows the achievement of new physicochemical properties [1-3] that lead to novel sensing platforms based, for instance, on electrical or optical transduction of chemical reactions [4-6].

The sizes of the one-dimensional (1D) nanostructures are comparable to those of the biological and chemical species. Therefore, they represent the excellent transducers for producing signals from the species being sensed. Among the variety of 1D nanostructures that have received considerable attention, silicon nanowires (NWs) demonstrated superior properties for the development of chemi- and biosensor families, e.g., DNA sensing devices, virus detectors, or pH sensors [7-9].

In these fields, silicon carbide nanostructures are also promising due to their physicochemical properties in photocatalysis [10], superplasticity [11], biocompatibility, and resistance to photobleaching [12]. In addition, recent works have demonstrated enhancement and possible tuning of the optical emission of silicon carbide NWs covered with native silicon dioxide amorphous shell [13,14]. This effect could be employed to improve the performances of sensors, based on the transduction of chemical reaction in optical emission, and also in possible *in vivo* application.

In this work, we describe a new class of fluorescent hybrid nanomaterials consisting of SiC/SiO_2_ core/shell nanowires functionalized with a model thiophene-based organic dye as potential biosensing candidate. The employment of SiC/SiO_2_ core/shell NWs has been decided to exploit well-known chemical methods for the functionalization of silicon dioxide, i.e., reaction with the oxydrilic group of the silica surface [15-17]. The oligothiophene component was selected because of the outstanding structural versatility, fluorescence, and charge-transporting properties of thiophene-based compounds that are among the most used organic materials for applications ranging from plastic electronics to biosensing and biodiagnostic [18,19]. Notably, oligothiophene compounds have been largely used for bio-imaging purposes and have shown high chemical and photochemical stability in biologic environment and non-toxicity for living cells [19,20]. The fluorophore (T3Pyr) selected for this work is characterized by a pyridine antenna, capable of cation complexation, and a triethoxysilane end moiety for the covalent binding of oxydrilated surfaces [21,22] such as the NW shell. This molecule has recently been used to realize fluorescent self-assembled monolayers with pH sensitive multicolor and bright fluorescence [23].

The synthesis of the 3C-SiC/SiO_2_/T3Pyr nanosystem and the morphological and optical characterization by combined scanning electron microscopy (SEM), transmission electron microscopy (TEM), and laser scanning confocal microscopy (LSCM) is herein described. The results show a homogeneous molecule grafting on the nanowire surface and the preservation, after the grafting, of the optical properties of the organic dye. This study can open new perspectives for applications of the novel nanostructured hybrid material as biosensing agent, combining the advantages of the surface-to-volume ratio of the SiC/SiO_2_ core/shell nanowires with the pH sensitive fluorescence properties of T3Pyr.

## Methods

**Figure 1:**
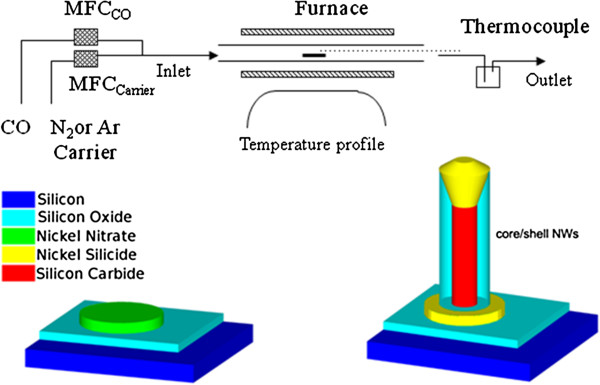
Schematic diagram of the experimental setup.

NW growth on Si (001) substrates was performed by a nickel-assisted carbothermal method, with flowing carbon oxide and nitrogen or argon as carrier gases in an open-tube configuration (see Figure 1).

Before being placed into the reactor, the substrate is cleaned in organic solvents with an ultrasonic bath, dipped in a 0.01-M solution of Ni(NO_3_)_2_ in ethanol, and dried in air (Figure 1: bottom left, colored figure). For the NW growth, the substrate is placed inside an open tube in the center of a horizontal furnace previously purged with inert gas to remove air. The temperature is raised to 1,100°C, and after temperature stabilization carbon oxide is introduced into the tube. The growth time of NWs selected for functionalization experiments is 30 min.

According to a preferential interface nucleation mechanism [24], dense networks of Ni-catalyzed SiC/SiO_2_ core/shell NWs are obtained via a vapor–liquid-solid growth process (Figure 1: bottom right, colored figure). In particular, the following different reactions could lead to the formation of the core/shell system:

(1) $$2\;\mathrm{CO}\left(g\right)+3\;\mathrm{Si}=2\;\mathrm{SiC}\left(s\right)+{\mathrm{SiO}}_{2}\left(s\right)$$

(2) $$\mathrm{CO}\left(g\right)+3\;\mathrm{SiO}=\mathrm{SiC}\left(s\right)+{2\;\mathrm{SiO}}_{2}\left(s\right)$$

The first has been proposed by Pongracz et al. to explain the growth of nanocrystals [25] and directly involves silicon from the substrate. The latter has been more often reported in the literature [26-28] for the growth of nanowires and involves SiO obtained by intermediate reactions between the substrate and carbon oxides.

The morphological and optical properties of the nanowires were investigated before functionalization by SEM and cathodoluminescence (CL) spectroscopy in a SEM Cambridge 360 Stereoscan (Cambridge Instruments Ltd., Cambridge, England) equipped with a Gatan MONO-CL2 spectrometer (Gatan, Inc., Pleasanton, CA, USA). Their structural and chemical properties were studied by TEM in a field-emission microscope (JEOL 2200FS, JEOL Ltd., Akishima, Tokyo, Japan) working at 200 kV, equipped with in-column omega filter, high angle annular dark field (HAADF) detector for Z-contrast experiments, and energy-dispersive X-Ray (EDX) microanalysis.

Fluorescence imaging was performed on an inverted Nikon A1 (Nikon Co., Shinjuku, Tokyo, Japan) laser scanning confocal microscope equipped with a 405-nm pulsed/CW diode laser (PicoQuant GmbH, Berlin, Germany). Confocal fluorescence imaging was carried out on the samples at 20°C. The 1,024 × 1,024-pixel images were collected using a Nikon PLAN APO VC 60 oil immersion objective with NA 1.40. With this imaging configuration, spatial resolution is *ca*. 210 nm in the x and y direction. Z-stack imaging was performed on 2.5-μm sections for a total thickness of 50 μm. Fluorescence was collected in the 500- to 550-nm spectral window.

Fluorescence lifetime imaging was performed, applying the time-correlated single-photon counting (TCSPC) technique. A single-photon avalanche diode (SPAD) detector was used for this scope. A 460- to 500-nm band-pass filter was placed in front of the operating SPAD. The instrument is integrated with PicoHarp 300 electronics (PicoQuant GmbH) for TCSPC measurements. The repetition rate of the pulsed excitation at 405 nm was 40 MHz. FWHM of the laser pulse is *ca*. 120 ps. A tail fit has been performed on the histogram calculated for a region of interest of the sample image. Fitting of the experimental trace to tri-exponential decay function yielded satisfactory results with a good chi-square value.

## Results and discussion

### Characterization before surface functionalization

**Figure 2:**
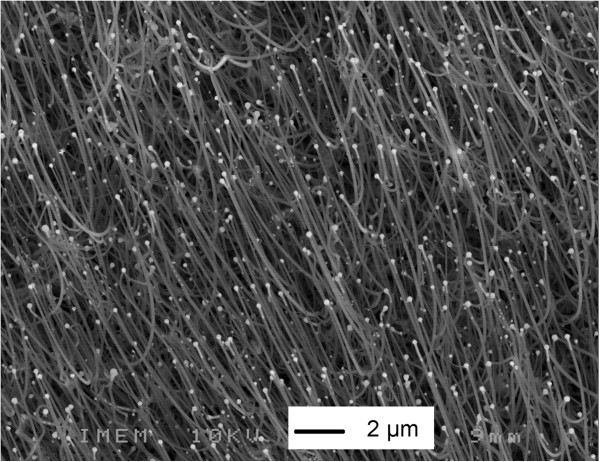
SEM image of the NW bundle before functionalization.

A secondary electron image of the as-grown SiC/SiO_2_ core/shell NWs is shown in Figure 2. The growth process gives bundles of wires with a length of several tens of microns and a diameter in the range of hundreds of nanometers. The NW growth takes place selectively inside the areas covered with the nickel-based catalyst so that it is possible to obtain on the substrate patterns with different geometries [29].

**Figure 3:**
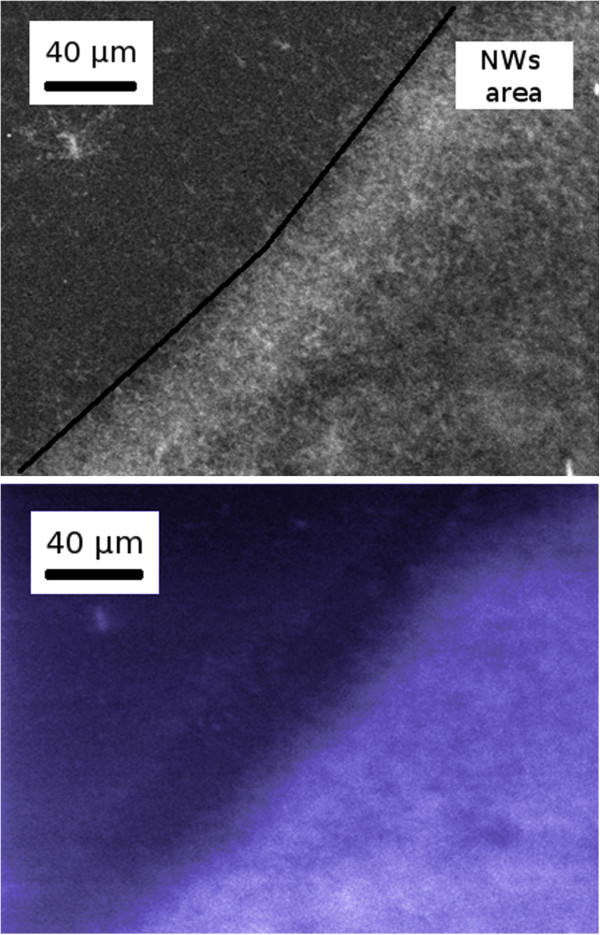
**SEM image and panchromatic CL image.** SEM image (top) and panchromatic CL image (false color, bottom) acquired at low magnification (×500) at the edge of the NW area. The black line is a guide for the eye along the edge of the NW area.

The comparison between the SEM image (Figure 3, top) and the corresponding CL panchromatic image (Figure 3, bottom) taken at the edge of an area in which the NW growth occurred demonstrates that the light emitted under electron excitation is localized only in the area covered by NWs.

**Figure 4:**
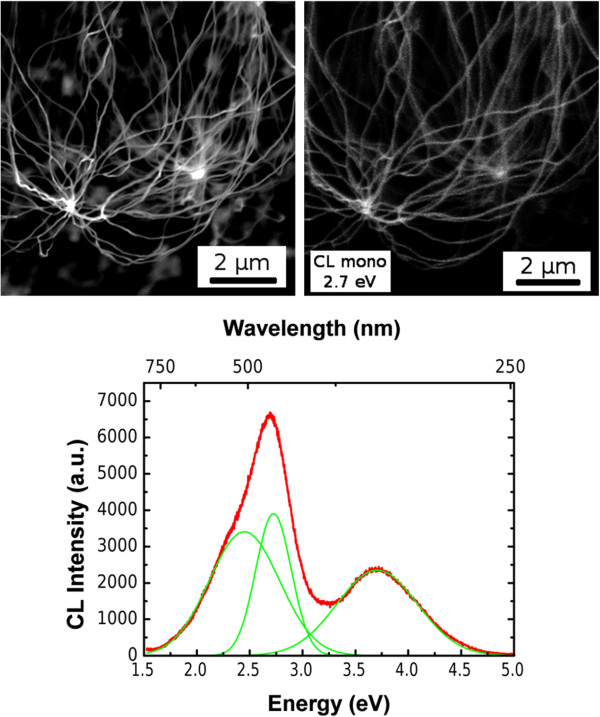
**SEM and corresponding monochromatic CL images and room-temperature CL spectrum.** Top image shows SEM (left) and corresponding monochromatic CL images acquired at 2.7 eV (right). Bottom image shows room-temperature CL spectrum taken on the same area.

A magnified SEM image of a region inside the NW bundle is shown in Figure 4 (top left). The room-temperature CL spectrum acquired in this region (Figure 4, bottom) presents two broad emission bands that resulted from three different components that peaked at 2.43 eV, related to the near-band-edge emission from the 3C-SiC core, and at 2.74 and 3.6 eV, involving the triplet and singlet states of oxygen deficiency centers (ODC(II)) [30] in the silicon dioxide shell. The CL monochromatic image taken at 2.7 eV (Figure 4, top right) shows a homogeneous distribution of the luminescence in the bundle and along the NWs. Thorough spectroscopic studies of the single nanowire emission, with the analysis of the line shape and the Gaussian deconvolution for NWs with different core-to-shell thickness ratio, are reported elsewhere [14].

**Figure 5:**
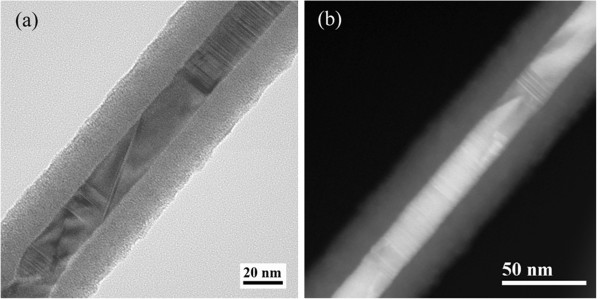
Typical zero-loss filtered image (a) and Z-contrast image (b) of the SiC/SiO_2_ NWs before functionalization.

 The core/shell geometry of the NWs and the crystalline structure of the core were examined by TEM. Figure 5 shows a typical zero-loss filtered image and a Z-contrast image, acquired on single NWs after HF etching of the sample in (1:3) aqueous solution for 60 s. As reported in a previous work [13], the etching acts selectively on silicon dioxide and allows the partial removal of the amorphous layer, leaving the core wrapped by the shell with a thickness ratio of about 1:1 compared to 1:5 of the as-grown sample [14]. This treatment is required for the TEM observation to enhance the diffraction contrast and improve the visibility of the inner core, allowing a study of its crystalline structure and defects. Perfect segments, unambiguously identified as 3C-SiC, alternate with defective regions, where the insertion of planar defects and in particular stacking faults on (111) planes perpendicular to the growth axis are found. In these regions, the occurrence of local stacking sequences of 2H, 4H, and 6H polytypes is observed, as commonly found in SiC whiskers [31,32]. As for the coaxial amorphous shell, which envelops the core in a cylindrical geometry, EDX analyses identified it as SiO_*x*_ layer, with *x* very close to 2.

### Surface functionalization

**Figure 6:**
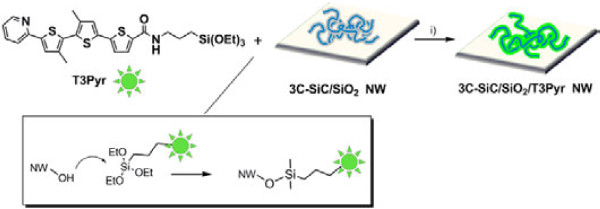
**Functionalization of NWs with T3Pyr.** (i) Toluene at room temperature for 24 h. The simplified reaction mechanism is depicted in the square.

The functionalization of SiC/SiO_2_ NWs with T3Pyr was achieved by dipping the NWs grown on a silicon substrate in a toluene solution of T3Pyr (C ≈ 10^−3^ M) for 24 h according to the sketch in Figure 6. After this time, the substrates were extracted, washed with fresh toluene/EtOH and acetone, and dried under vacuum. The covalent binding of T3Pyr fluorophores to the NW surface occurs by the reaction of the OH moieties of the NW silica shell and the triethoxysilane groups of T3Pyr as shown in the inset of Figure 6.

### Characterization after surface functionalization

**Figure 7:**
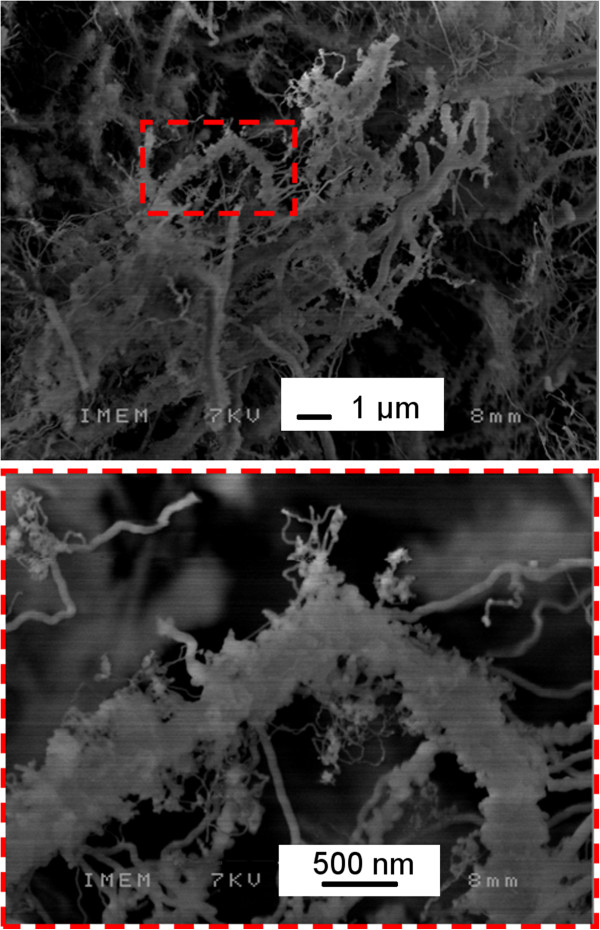
**SEM image of the functionalized NWs.** The area marked in red is enlarged on the bottom panel. Micrometric-sized features are seen due to the aggregation of NWs.

The functionalized NWs were characterized by SEM, TEM, and LSCM techniques. SEM images of functionalized NWs are presented in Figure 7. The comparison between the as-grown NWs (Figure 2) and the functionalized NWs demonstrates that the functionalization process induces an aggregation of NWs up to a micrometric size. This effect is most likely related to the aggregation of fluorophores.

**Figure 8:**
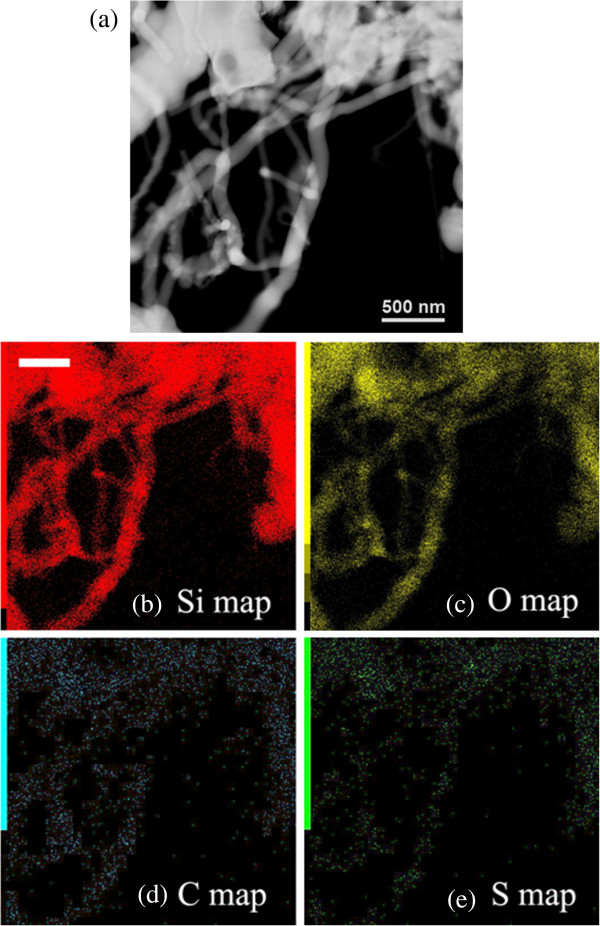
**HAADF image and EDX elemental maps.** (**a**) HAADF image and (**b**, **c**, **d**, **e**) EDX elemental maps acquired in the same area in scanning mode with a probe size of about 1 nm. Scale bar is 500 nm; each map is obtained from the K-edge signal of the selected element.

Figure 8 shows the Z-contrast image (Figure 8a) and the corresponding EDX elemental maps of silicon, oxygen, carbon, and sulfur (Figure 8b,c,d,e). Since the wires are SiC/SiO_2_ with a thick shell, the signal-to-noise ratio for Si and O is higher than for C and S, but the spatial distribution of each element resembles the NW geometry, indicating that the signal comes from the wires. In particular, the carbon distribution is more spread than it would be, considering only the SiC core size, suggesting a main contribution from C of the oligothiophene framework. Further, the sulfur signal found along the treated wires is not detected in the as-grown samples. These results demonstrate the positive occurrence of surface functionalization with T3Pyr molecules.

**Figure 9:**
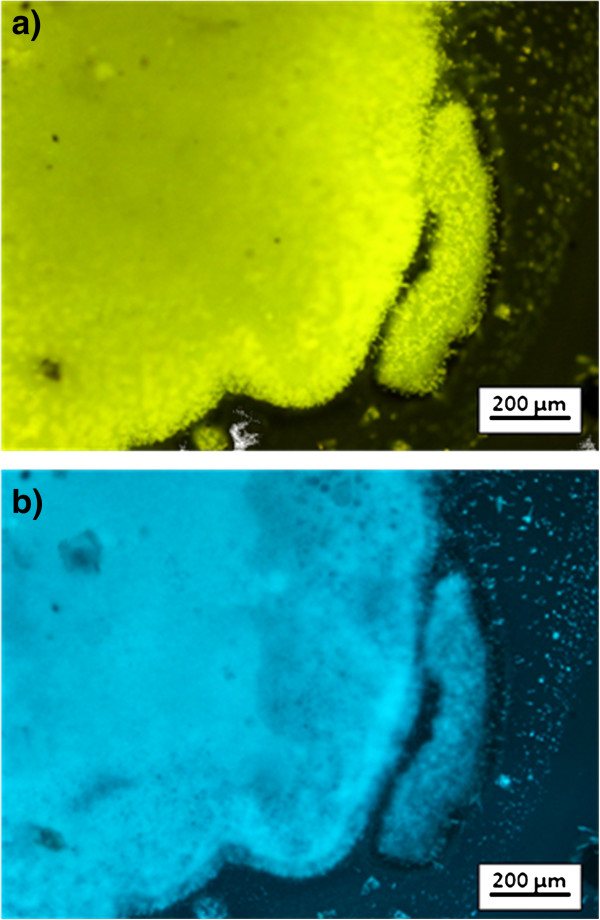
**Fluorescence microscopy images of T3Pyr functionalized NWs.** They are treated with HCl/EtOH solution at pH 3 (**a**) and then with triethylamine at pH 8.5 (**b**).

Evidence of surface functionalization comes also from the treatment of T3Pyr functionalized NWs by acid (HCl/EtOH) or basic solution (triethylamine, EtOH). Fluorescence microscopy (FM) analysis shows a clear fluorescence change on the changing surface pH by casting a drop (5 μL) of acid/basic solution. Similar to what is observed for T3Pyr in solution and self-assembled monolayers [23], a switch of the fluorescence emission from blue to yellow can be seen (Figure 9).

**Figure 10:**
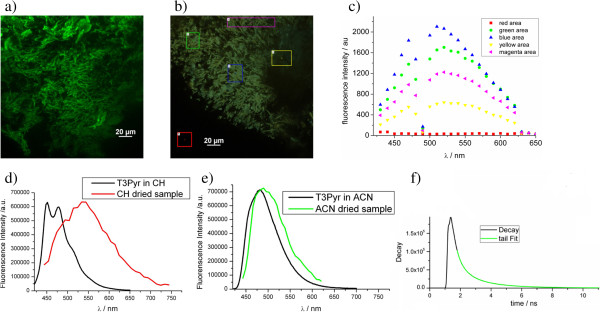
**Maximum intensity projection of Z**-**stack, fluorescence confocal image, various fluorescence spectra, and tri-exponential fitting.** (**a**) Maximum intensity projection of Z-stack for section thickness of 50 μm; 500- to 550-nm single-band band-pass filter. (**b**) Fluorescence confocal image of T3Pyr-functionalized nanowires for excitation at 405 nm. (**c**) Fluorescence spectrum of the five different areas evidenced in (**b**), obtained with a Nikon A1 spectral module using a 10-nm resolution. (**d**, **e**) Fluorescence spectrum of T3Pyr in solution. (**f**) Tri-exponential fitting of the decay calculated for an area of interest in (**a**), yielding lifetimes of 0.3, 1.0, and 2.3 ns.

Accordingly to the SEM and FM results, the LSCM shows bundles of wires with a length of several tens of microns and a diameter of about 2 μm. The maximum intensity projection of Z-stack images for a sample thickness of 50 μm evidences clearly the nanowires of several tens of micrometers (see Figure 10a). We obtained spectral fluorescence images of the nanowires (see Figure 10b). The fluorescent spectrum (Figure 10c) resembles that obtained for T3Pyr in solution, even though relative fluorescence intensity above 500 nm is higher compared with the fluorophore emission in solution (see Figure 10d,e), confirming a homogeneous functionalization of the nanowires. The more pronounced shoulder above 500 nm may be due to the close proximity of fluorophores on the nanowires leading to excimer fluorescence. The latter has been reported with a red-shifted spectrum compared to the monomeric fluorescence and a lifetime of 4.2 ns compared to 0.35 ns reported for single-fluorophore emission [33]. We also performed time-correlated single-photon counting measurements exciting at 405 nm in order to obtain fluorescence lifetime images. Global fitting with a tri-exponential function (see Figure 10f) yielded a satisfactory chi-square of 1.2. The lifetimes are 0.3, 1.0, and 2.3 ns. Homogeneous distribution of the lifetimes along the wires was observed. Plotting the image as a function of the intensity ratio of two lifetimes, it is clear that the species with a lifetime of 2.3 ns gives the most important contribution to the fluorescence of the functionalized nanowires. Time-resolved fluorescence confocal imaging for an ACN or CH T3Pyr sample in ACN or CH dried on a glass cover slip also yielded more than one lifetime: a short dominating one of 0.3 ns and a longer one of 1.4 and 0.9 ns. Likely, the short lifetime is similar to the fluorescence lifetime of monomeric T3Pyr in ACN or CH solution which is in agreement with literature data [34], while aggregation of fluorophores gives rise to excimer fluorescence with a longer lifetime, and this holds also for the functionalized NWs.

## Conclusions

A new nanostructured hybrid material is developed by direct grafting of a model thiophene-based organic dye on 3C-SiC/SiO_2_ core/shell nanowire surface. A structural characterization carried out on the as-grown nanostructures reveals that the nanowires are a 3C-SiC core wrapped in a quasi-stoichiometric silicon dioxide. The cathodoluminescence spectroscopy presents two broad emission bands that resulted from three different components that peaked at 2.43 eV, related to the near-band-edge emission from the 3C-SiC core, and at 2.74 and 3.6 eV, involving the triplet and singlet states of ODC(II) in the silicon dioxide shell. TEM-EDX analysis of the functionalized nanowires reveals that the carbon distribution is more spread than it would be considering only the SiC core size, suggesting a main contribution from C of the oligothiophene framework. Further, a sulfur signal not detected in the as-grown samples is found along the treated wires. In addition, the confocal fluorescent spectroscopy shows similar line shapes of functionalized NWs and T3Pyr in solution, confirming homogeneous molecule grafting on the nanowire surface, without any influence of the luminescence properties of the inorganic component. Chemical and luminescence characterizations confirm a homogeneous functionalization of the nanowires; in particular, the organic dye maintains its optical properties after the grafting, thus opening a possible perspective of this new nanostructured hybrid material in the field of chemo-/biosensing applications.

## Competing interests

The authors declare that they have no competing interests.

## Authors' contributions

FF and FR conceived this study, carried out the SEM, SEM-CL, and TEM characterizations, and drafted the manuscript. MM performed the functionalization with T3Pyr and treatments of functionalized NWs. IM performed the confocal fluorescence studies. GA grew the NWs. LF and MZ participated in the sample preparation and fluorescence imaging. GS participated in the coordination of the study and revised the manuscript. All the authors discussed the results and contributed to the final version of the manuscript. All the authors read and approved the final manuscript.
